# Visual outcomes in a series of patients with papilledema and comorbid nutritional deficiency

**DOI:** 10.3389/fopht.2025.1705302

**Published:** 2026-01-15

**Authors:** Dan V. Spiegelman, John Burkland, Evan Jameyfield, Gregory P. Van Stavern, Leanne Stunkel

**Affiliations:** 1 Washington University in St. Louis School of Medicine, St. Louis, MO, United States; 2 Department of Ophthalmology and Visual Science and Department of Neurology, Yale School of Medicine, New Haven, CT, United States; 3 John F. Hardesty, Medical Doctor (MD) Department of Ophthalmology and Visual Sciences and Department of Neurology, Washington University in St. Louis, St. Lous, MO, United States

**Keywords:** papilledema, IIH, micronutrient deficiencies, nutritional optic neuropathy, satisfaction of search error

## Abstract

**Background:**

Fulminant papilledema is a neuro-ophthalmologic emergency that can lead to permanent vision loss as a result of optic neuropathy. Despite appropriate treatment of intracranial hypertension and improvement of papilledema, some patients continue to experience worsening vision. In some cases, comorbid micronutrient deficiencies, which are an additional cause of optic neuropathy, may contribute to this decline. This case series highlights patients with papilledema whose vision worsened despite improvement in papilledema and who were found to have comorbid nutritional deficiencies.

**Methods:**

A retrospective case series of three patients with papilledema were seen between 2019 and 2022 at Washington University in St. Louis and Barnes-Jewish Hospital. All experienced continued vision loss despite successful treatment of intracranial hypertension and were subsequently also diagnosed with nutritional deficiencies. Data on Body Mass Index (BMI), visual acuity, papilledema grade, optical coherence tomography findings, visual fields, and treatment outcomes were analyzed.

**Results:**

These three female patients had worsening vision despite medical and/or surgical treatment for intracranial hypertension. All were found to have comorbid nutritional deficiencies (thiamine, B12, folate, and/or copper). Following appropriate nutritional supplementation, none experienced continued worsening of their visual function.

**Conclusions:**

Worsened vision loss despite papilledema resolution may occur in patients with comorbid nutritional deficiencies. Although we cannot prove a causative relationship with our case series, it may be worth considering screening for micronutrient deficiencies in at-risk patients, as these deficiencies can have visual consequences and are straightforward to treat.

## Introduction

Fulminant papilledema is a neuro-ophthalmologic emergency that can lead to permanent vision loss due to optic nerve damage. It is an uncommon presentation of idiopathic intracranial hypertension (IIH), usually affecting young women who are obese (1), or a consequence of secondary causes of intracranial hypertension, like meningitis or dural venous sinus thrombosis. In some cases, patients whose papilledema is appropriately treated have worsening of visual function despite improvement in papilledema (2, 3).

Micronutrient deficiencies can also cause vision loss from optic neuropathies. These typically involve changes in visual acuity, color vision, or central/cecocentral scotomas and are associated with deficiencies of thiamine, folate, vitamin B12, and copper (4–7). Importantly, obesity does not exclude the possibility of micronutrient deficiencies (8).

Neuro-ophthalmic conditions are complex and have been shown to be vulnerable to diagnostic errors like “search satisfaction”, the tendency to stop searching once one cause for a condition has been identified (9–11). In patients with papilledema, providers may not consider the possibility that patients may have comorbid optic neuropathies, potentially leading to worsened visual outcomes.

Here, we describe three cases of patients with papilledema and vision loss involving the central vision, whose vision worsened despite improvement of their papilledema, and who were found to have comorbid nutritional deficiencies. It is possible that the nutritional deficiencies may have been an additional contributor to the optic neuropathy in these patients, contributing to worsening visual outcome.

## Materials and methods

A retrospective case series of three patients were seen between 2019 and 2022 at Washington University in St. Louis and Barnes-Jewish Hospital. Medical history, surgical interventions, pharmacologic treatments, vitamin supplementation, and visual outcomes (including visual acuity, perimetric mean deviation, papilledema grade, retinal nerve fiber layer, and ganglion cell complex thicknesses) were recorded. Optical coherence tomography (OCT) measurements were performed using a Zeiss Cirrus scanner. Patient identifiers were destroyed at the earliest opportunity.

## Results

Demographic and clinical data are summarized in **Table 1**.

Table 1Clinical characteristics of patients.

**Table:** 

Patient Case	BMI	Surgical intervention for papilledema (indication)	Nutritional deficiency	Nutritional chemistry after supplementation	Cause of nutritional deficiency	Hematology before supplementation	Hematology after supplementation	Supplementation type, dose, and duration	Nadir visual acuity	Final visual acuity	Nadir Mean deviation from visual fields	Final mean deviation	Peak average RNFL (μm) and papilledema grade (Frisén Scale)	Final average RNFL (μm) and GCC (μm)
1	32.9	VP shunt (worsening despite max-dose)	B1: 62 nmol/L	B1: 86 nmol/L [70–180]	Presumed alcohol use	White Blood Cells (WBC): 3.5 K/cumm	WBC: 3.8 K/cumm	100 mg B1 daily, 1,000 μg B12 daily for 10 months	HM OD	HM OD	−33.79 OD, −30.48 OS	Stimulus Size (STIM) 5 OD, STIM 5 OS	224 OD, 153 OS	RNFL: 60 OU
			B12: 195 pg/mL MMA: 0.08 nmol/mL	B12: 467 pg/mL [230–1,250]		Hemoglobin (Hgb): 7.3 g/dL	Hgb: 14.1 g/dL		20/25 OS	20/20 OS			Grade 3 OU	
				MMA: 0.07 nmol/mL [<0.40]		Hematocrit (Hct): 26.5%	Hct: 42.2%							Ganglion Cell Complex (GCC): 48 OD, 51 OS
						Platelets (Plt): 570 K/cumm	Plt: 296 K/cumm							
2	35.55	None	B12: 191 pg/mL	No repeat labs	Unknown	WBC: 10.0 K/cumm	WBC: 11.4 K/cumm	1,000 μg B12 daily for	20/20 OD	20/20 OU	−29.58 OD	−4.21 OD,?OS	223 OD, 219 OS	RNFL: 67 OD, 48 OS
			MMA: 0.32 nmol/mL			Hgb: 11.7 g/dL	Hgb: 14.6 g/dL	11 months	20/60 OS		−7.41 OS			GCC: 74 OD, 53 OS
						Hct: 34.7%	Hct: 45.8%					Grade 3 OU	Grade 3 OU	
						Plt: 252 K/cumm	Plt: 216 K/cumm							
3	30	VP shunt (fulminant IIH in context of medication non-adherence)	B1: 52 nmol/L	B1: 215 nmol/L [70–180]	Unknown	WBC: 6.1 K/cumm	WBC: 3.9 K/cumm	100 mg B1 daily for 2 months	HM OD	HM OD	Unable to obtain due to level of vision loss	Unable	194 OD, 256 OS	RNFL: 62 OD, 59 OS
						Hgb: 16.6 g/dL	Hgb: 12.1 g/dL		NLP OS	NLP OS			Grade 3 OU	GCC: 38 OD, 52 OS
						Hct: 25%	Hct: 35.5%							
						Plt: 239 K/cumm	Plt: 193 K/cumm

**Table:** Micronutrient normal values supporting legend.

Micronutrient	Normal values
B1	70–180 nmol/mL
Folate	≥5 ng/mL
Cu	73–129 μg/dL
B12	230–1,250 pg/mL
MMA	<0.40 nmol/mL

RNFL, retinal nerve fiber layer; VP, ventriculoperitoneal; MMA, methylmalonic acid; HM, hand motion; OD, right eye; OS, left eye; OU, both eyes; IIH, idiopathic intracranial hypertension.

.

### Case 1

A woman aged 30–35 with BMI of 32.9 kg/m^2^, medical history of IIH diagnoses 10 years prior and treated with diuretic (unclear specific medication), and a diagnosis of iron deficiency anemia 2 months prior presented with recurrent grade 3 papilledema in the right eye (OD), grade 2 papilledema with pallor in the left eye (OS), and OCT retinal nerve fiber layer (RNFL) 224 μm OD, 153 μm OS. At the time of presentation, pinhole corrected distance visual acuity (dVAph) was 20/50 OD, 20/25 OS, and 7/11 color plates OD, 10/11 OS, with Humphrey visual field (HVF) showing generalized visual field constriction and depression in both eyes (OU) (**Supplementary Figure 1A**). Magnetic resonance imaging (MRI) of the brain and orbits and magnetic resonance venography (MRV) of the head with and without contrast were normal, other than radiographic signs of intracranial hypertension. Lumbar puncture (LP) measured opening pressure (OP) greater than 55 cm H_2_O in the lateral decubitus position, with bland cerebrospinal fluid (CSF) contents.

Despite maximum dose acetazolamide (4 g daily), her vision deteriorated over the next 4 weeks to dVAph 20/300 OD, 20/25 OS, no control color plate OD, and 8.5/11 color plates OS. Visual fields had an interval worsening of generalized depression (**Supplementary Figure 1B**). She declined emergency room and inpatient care multiple times, but within 2 weeks, she underwent successful ventriculoperitoneal (VP) shunt placement, 6 weeks after her initial presentation.

She was seen in follow-up examination less than 2 weeks after VP shunt placement and was noted to have interval resolution of her papilledema; OCT RNFL had decreased to 84 μm OD, 74 μm OS, but her visual function had worsened to uncorrected distance visual acuity (dVAsc) bare hand motion (HM) OD, 20/25 OS, and no control plate OD, 3/11 OS, with worsened generalized constriction and generalized depression OD>OS on HVF (**Supplementary Figure 1C**). A shunt evaluation showed a working shunt with no shunt malfunction, and laboratory work-up revealed comorbid thiamine and B12 deficiencies, with thiamine level 62 nmol/L, B12 level 195 pg/mL, and methylmalonic acid (MMA) 0.07 nmol/mL in the setting of heavy alcohol use. Alcohol use is often seen to be correlated with nutritional deficiencies that may cause optic neuropathy, but alcohol is not thought to be directly toxic to the optic nerve (12).

The nutritional deficiencies were treated with supplementation, with subsequent laboratory-confirmed normalization of her nutritional levels. Her visual function stabilized at dVAsc HM OD, 20/20 −2 OS, and no control plate OD, 4.5/11 color plates OS. Her visual field performance did not worsen (**Supplementary Figure 1D**). She reported subjective improvement in overall visual function.

### Case 2

A woman aged 45–50, with a BMI of 36 kg/m^2^ and a medical history of type II diabetes mellitus complicated by retinopathy, hypertension, and hyperlipidemia was found to have fulminant papilledema secondary to aseptic meningitis. At presentation, she had pinhole corrected near visual acuity (nVAph) 20/20 OD, 20/30, 11/11 color plates OU, and generalized constriction to confrontation visual field testing OS>OD, with only a “very small central island” OS preserved, and grade 4 papilledema with hemorrhages OU. MRI and MRV of the head without and with contrast were normal, other than radiographic signs of intracranial hypertension. LP measured OP 45 cm H_2_O in the prone position with greater than 100 nucleated cells with a lymphocytic predominance.

She was treated with the maximum dose of acetazolamide (4 g daily). At a clinic follow-up 1 week after discharge, she demonstrated dVAsc 20/20 OD, ph 20/30 OS, and 14/14 color plates OU, with formal visual field testing showing constriction OS>>OD (**Supplementary Figure 2A**), with some improvement of her papilledema to grade 4 OD, grade 3 OS, with OCT RNFL 223 μm OD, 219 μm OS. She continued to demonstrate improvement in papilledema to trace papilledema and OCT RNFL 80 μm OD, 60 μm OS with stable visual acuity and improvement in visual field performance (**Supplementary Figure 2B**).

However, approximately 5 months after her initial presentation, her vision worsened to Pinhole Visual Acuity with Correction (VAccph) 20/20 OD, 20/60 OS, 10/11 color plates OD, 2.5/11 color plates OS, with worsened generalized constriction and depression of visual fields OS>OD (**Supplementary Figure 2C**) despite the resolution of the papilledema. Repeat LP measured OP 17 cm H_2_O in the lateral decubitus position. She was found to have B12 deficiency [191 pg/mL (230–1,250); MMA, 0.32 nmol/mL (<0.40)], which was treated with supplementation. B12 level improved with supplementation. At a follow-up 11 months later, her visual acuity stabilized to dVAph 20/20 OU with 11/11 color plates OD and 9.5/11 color plates OS. Her visual field performance did not worsen (**Supplementary Figure 2D**). She reported subjective improvement in visual function.

### Case 3

A woman aged 35–40 with a BMI of 30 kg/m^2^, a complex medical history significant for a previous history of IIH diagnosed 6 years prior and controlled with acetazolamide, and a history of end-stage renal disease (ESRD) on intermittent hemodialysis presented with 1 month of progressively worsening blurred vision. Examination showed distance VAsc 20/200 OD with no improvement with pinhole correction (phni), light perception OS, with grade 3 papilledema and gliosis bilaterally, and OCT RNFL 294 μm OD, 196 μm OS. She was unable to perform formal visual field testing due to her level of vision loss.

She was admitted for urgent treatment. MRI of the brain and orbits and MRV of the head without contrast (contrast was contraindicated due to ESRD) was normal, other than radiographic signs of intracranial hypertension. LP measured OP 53 cm H_2_O in the lateral decubitus position with bland CSF contents, and she received a VP shunt 6 days after that initial examination. Unfortunately, the VP shunt was complicated by intraparenchymal hemorrhage and subarachnoid hemorrhage in the left frontal lobe (not affecting the visual pathways) and required revision.

Vision continued to worsen after successful shunt revision to best corrected visual acuity (BCVA) at distance Count Fingers (CF) at 1 ft. OD, No Light Perception (LP) OS, despite an interval decrease in the RNFL thickness to 66 μm OD, 86 μm OS. LP measured OP 34 cm H_2_O, and the shunt was adjusted. At that time, further laboratory work-up revealed thiamine deficiency of 52 nmol/L with no obvious cause. Thiamine supplementation was started, with stabilization of her visual acuity and laboratory-confirmed normalization of her serum thiamine level. Four months later, she was seen for follow-up with subjective worsening of vision after she had been told to stop the thiamine supplement at the time of discharge after an unrelated hospitalization. At that time, she was found to have recurrent thiamine deficiency to 51 nmol/L, which was again treated with supplementation. After 2 months of supplementation, her subjective visual function stabilized. Her distance visual acuity remained VAsc HM OD, NLP OS.

## Discussion

Patients with fulminant papilledema are at risk for permanent vision loss due to optic nerve damage. Occasionally, patients continue to have worsening vision despite the apparent success of acute interventions. Providers may assume that new visual decline is related to the process underlying the fulminant papilledema and may not evaluate for comorbid contributing causes to optic nerve damage. In some cases, like those we present here, patients may have both fulminant papilledema and a comorbid condition like nutritional optic neuropathy, which could affect overall visual outcome.

This case series includes three patients with papilledema found to have comorbid nutritional deficiencies. In each case, intracranial hypertension was successfully treated, and there was improvement of the papilledema, but the patient’s visual function continued to worsen. After the nutritional deficiencies were corrected, visual function stabilized (no further worsening).

Optic nerve damage can be multifactorial. Typical findings of nutritional optic nerve damage may be masked by the presence of comorbid optic disc swelling. Nutritional optic neuropathy typically presents with bilateral, progressive cecocentral vision loss, while papilledema classically causes enlargement of the blind spot, but, when severe, can also cause defects in the central visual field (12, 13). On fundoscopic examination, nutritional optic neuropathy classically appears as a pale, swollen, or hyperemic optic disc; swelling of the optic disc due to papilledema would be expected to mask these signs (12, 13). We do not suggest that the micronutrient deficiencies contributed to the development of papilledema (14, 15). Instead, we explore whether some patients with fulminant papilledema may have micronutrient deficiencies that could contribute to the overall worsening of their optic neuropathy. Micronutrient deficiencies can have serious short- and long-term neurologic and visual consequences and are straightforward to treat. Obesity, a risk factor for IIH, does not preclude patients from being malnourished, as micronutrient deficiencies have been described in patients with elevated BMI (8). Although it may be counterintuitive, clinicians should not exclude the possibility of a micronutrient deficiency in an obese patient.

Limitations of this case series include its small size and retrospective design. In some cases, the patient’s low level of vision or inpatient status limited examiners’ ability to obtain formal distance vision or formal perimetry at every evaluation, and different examiners may have examined the patient at different times. With these cases, we cannot prove a causal relationship—we cannot know whether the visual outcome for these patients would have been worsened if their comorbid nutritional deficiency was never identified and treated, or whether it could have been improved if their nutritional deficiencies had been identified earlier in their course, and it is not possible based on this case series to prove that the micronutrient deficiencies contributed to worsening of the patients’ optic neuropathy. We acknowledge that worsening visual function even as papilledema resolves has been described (2, 3) and does not necessarily require the identification of a comorbid condition.

However, in the cases we present here, our clinical assessment of their course was that their papilledema was improving due to successful treatment and not due to axonal loss, and these patients were found to have comorbid micronutrient deficiencies that may have contributed to the visual deficits. In some cases, clinicians may consider expanding the search for additional contributors to visual dysfunction in order to avoid missing an additional/contributing factor that has the potential to contribute to a poorer visual outcome. Additionally, patients with compromised optic nerve function, like those in this study, are at risk for further progressive visual loss. Alterations to the normal architecture of the nerve and electrophysiological changes from the original insult can complicate the assessment of these patients, and it is important to protect them from other issues that may worsen their visual outcome.

In patients with progressive central vision changes, providers may consider evaluating for comorbid micronutrient deficiencies even when there are other known causes, such as fulminant papilledema. There is minimal risk to screening for and treating these conditions, and they may contribute to worsened visual outcomes, as well as other systemic symptoms, without intervention.

## Data Availability

The original contributions presented in the study are included in the article/**Supplementary Material**. Further inquiries can be directed to the corresponding author.

## Supplementary material

Supplementary Figure 1 Case 1: **(A)** Initial presentation Humphrey visual field (HVF) 24-2 size 3 stimulus both eyes (OU). **(B)** 2 weeks prior to ventriculoperitoneal (VP) shunt. HVF 24-2 size 3 stimulus OU. **(C)** Less than 2 weeks after VP shunt. HVF 24-2 size 3 stimulus OU. **(D)** Most recent visual field, obtained 3 years after initial presentation, with size 5 stimulus OU.

Supplementary Figure 2 Case 2: **(A)** Initial Humphrey visual field (HVF) 24-2 size 3 stimulus both eyes (OU) obtained 1 week after hospital discharge. **(B)** HVF 24-2 with size 3 stimulus OU obtained 1 month after hospital discharge. **(C)** Worsening visual fields despite improved papilledema, HVF 24-2 size 3 stimulus OU. **(D)** Most recent visual fields available, obtained around 1 year after initial presentation. HVF 24-2 with size 3 stimulus in right eye but size 5 stimulus in left eye.
