# Supplementary material for: Visual outcomes in a series of patients with papilledema and comorbid nutritional deficiency
Source: Front Ophthalmol (Lausanne). 2026 Jan 15;5:1705302. doi: 10.3389/fopht.2025.1705302 (PMC12851949; doi:10.3389/fopht.2025.1705302)

Supplementary Figure 1: Case 1

A. Case 1. Initial presentation Humphrey visual field (HVF) 24-2 size 3 stimulus both eyes (OU).


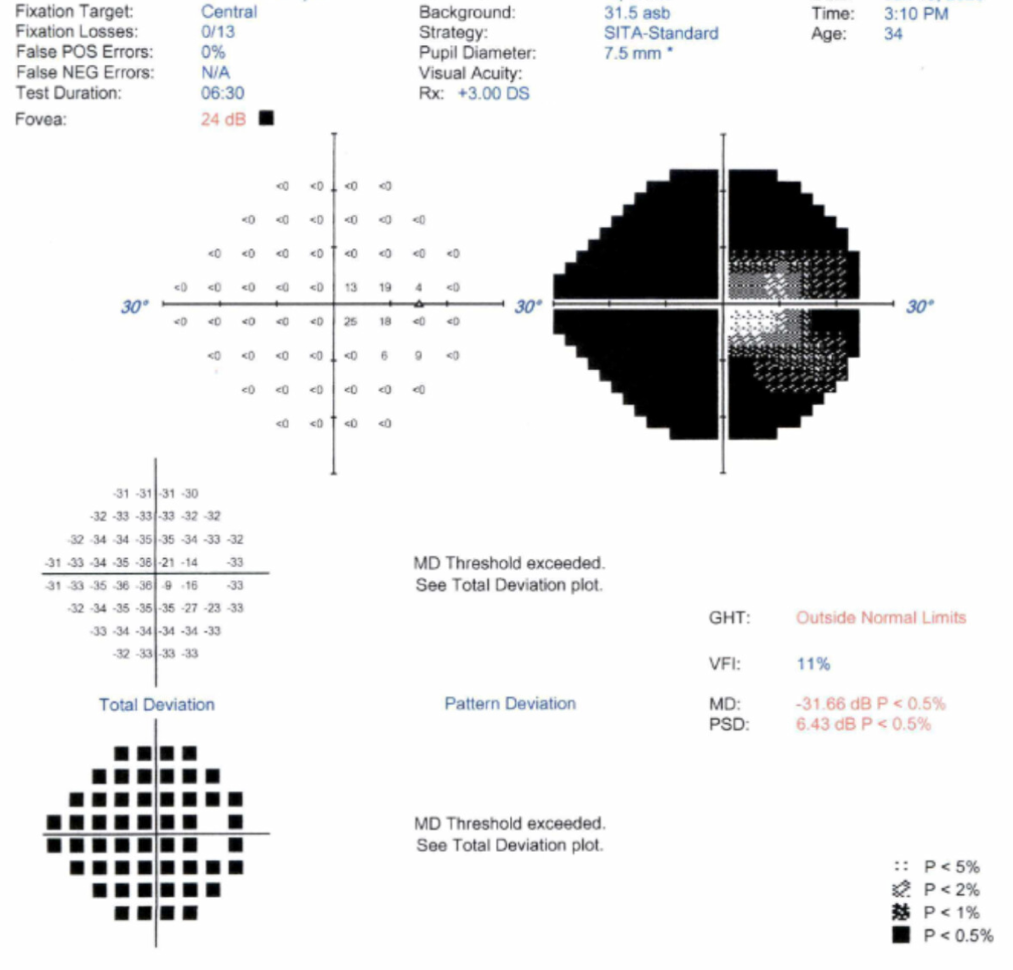

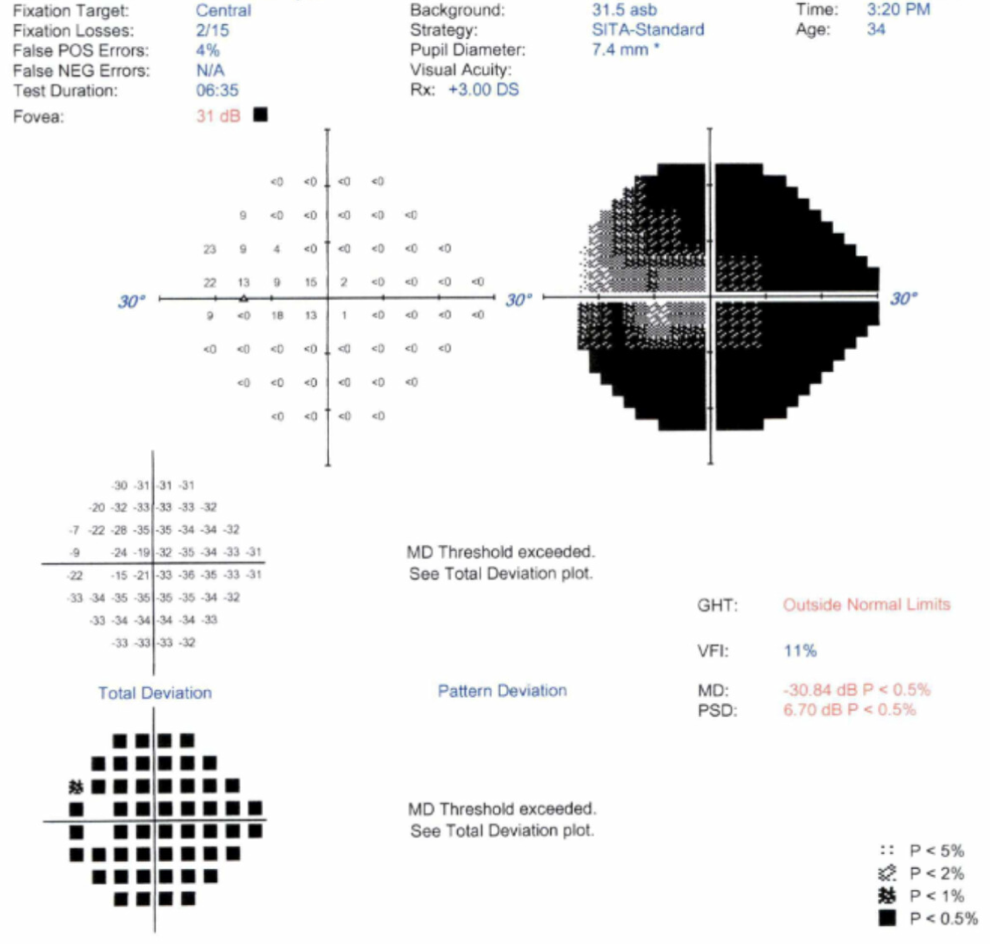


B. Case 1. 2 weeks prior to ventriculoperitoneal (VP) shunt. HVF 24-2 size 3 stimulus OU.


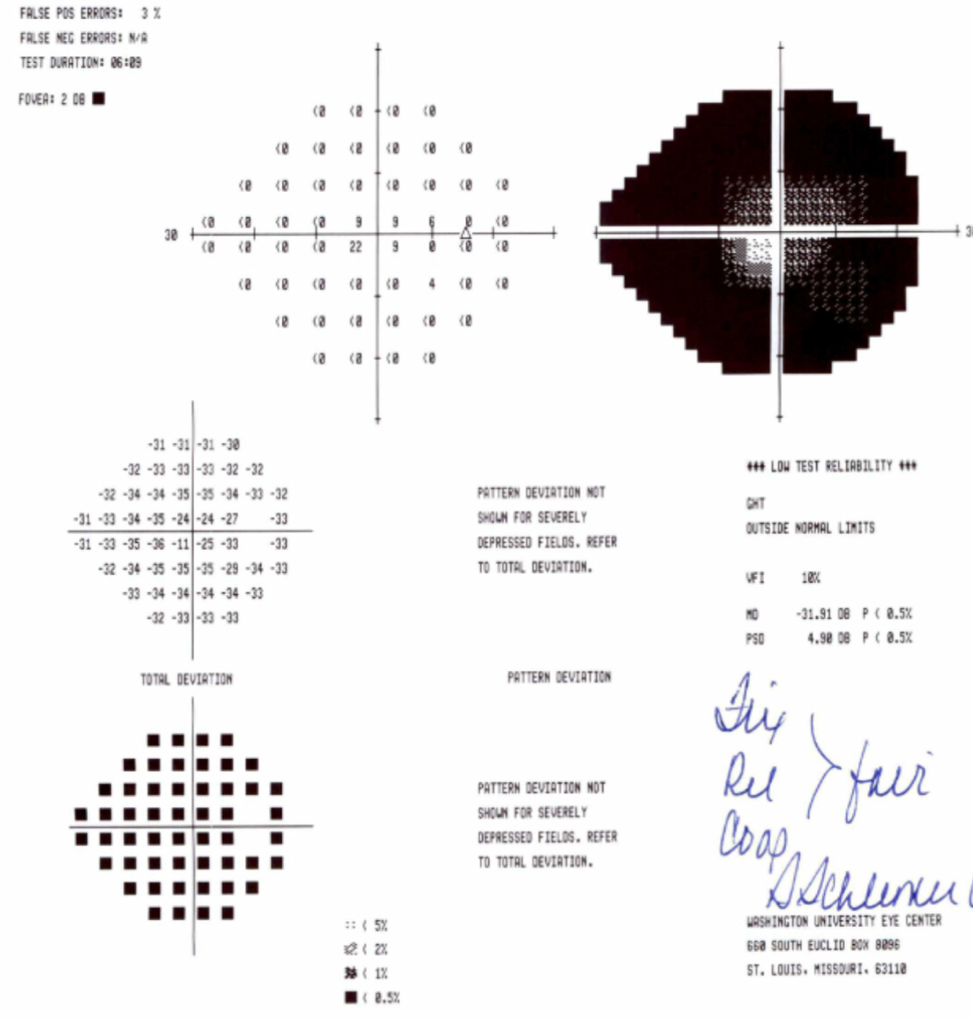

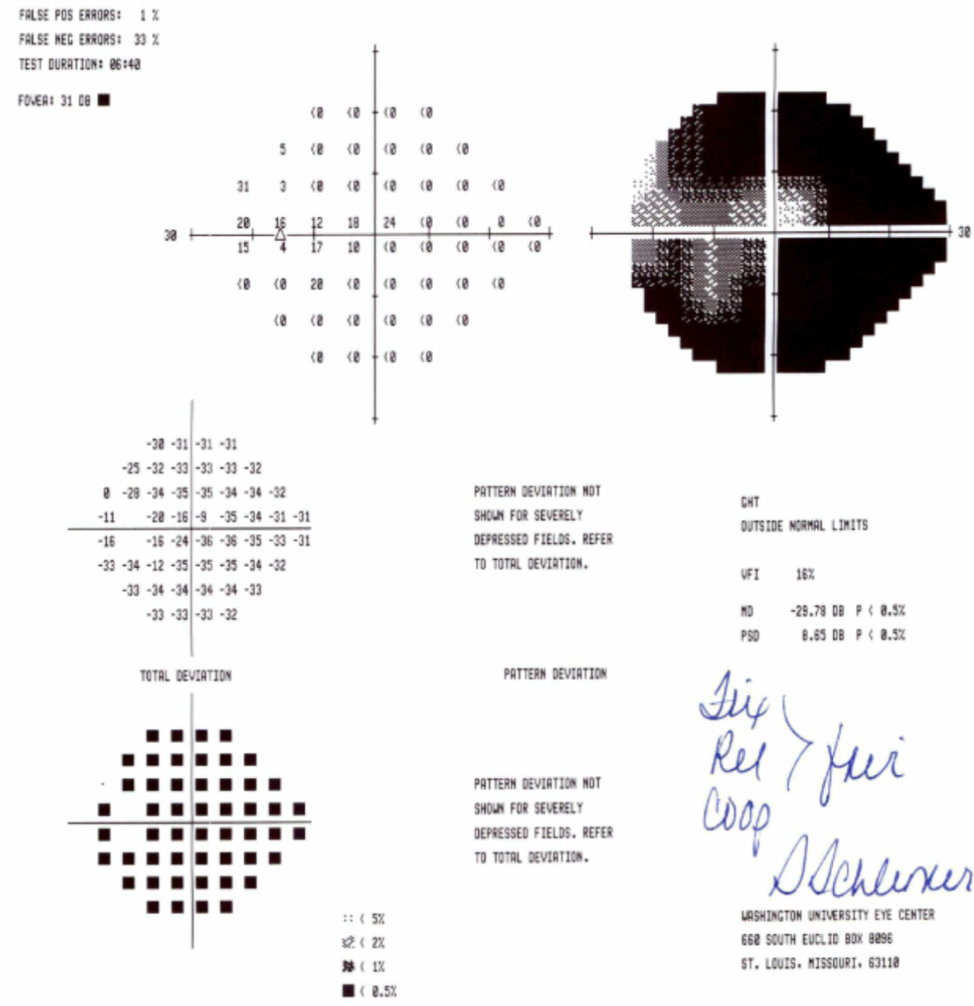


C. Case 1. Less than 2 weeks after VP shunt. HVF 24-2 size 3 stimulus OU.


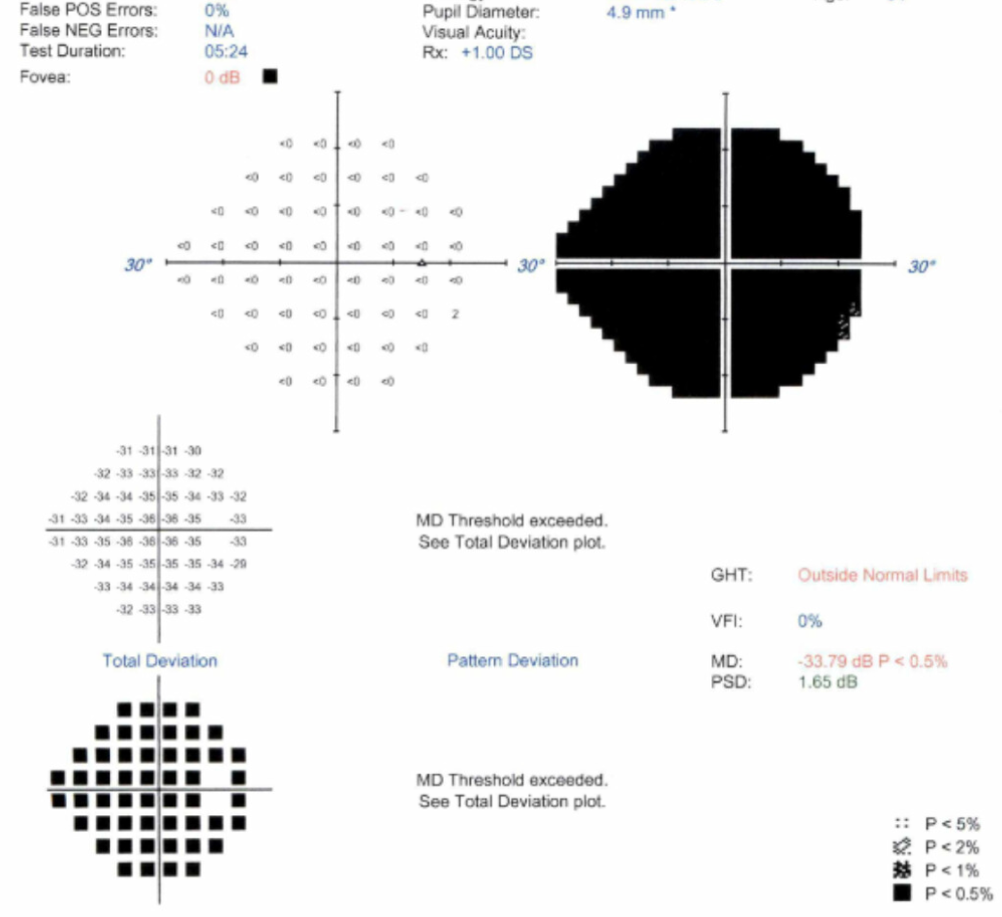

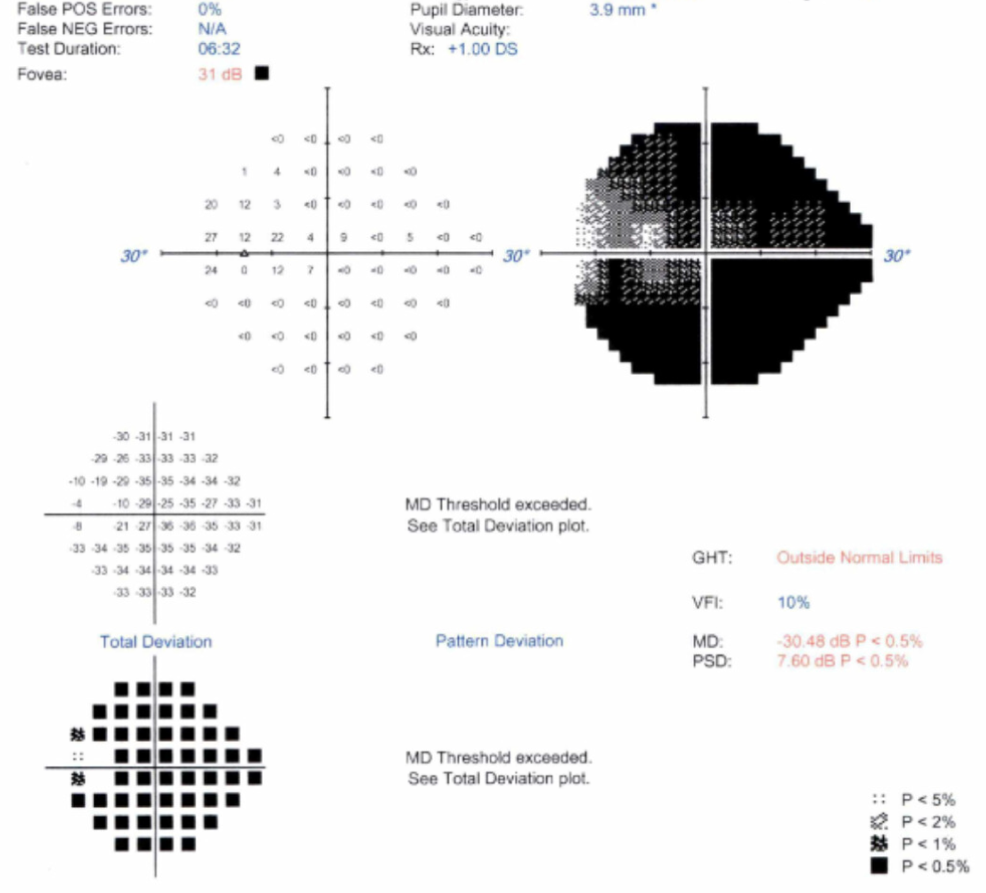


D. Case 1. Most recent visual field, obtained 3 years after initial presentation, with size 5 stimulus OU.


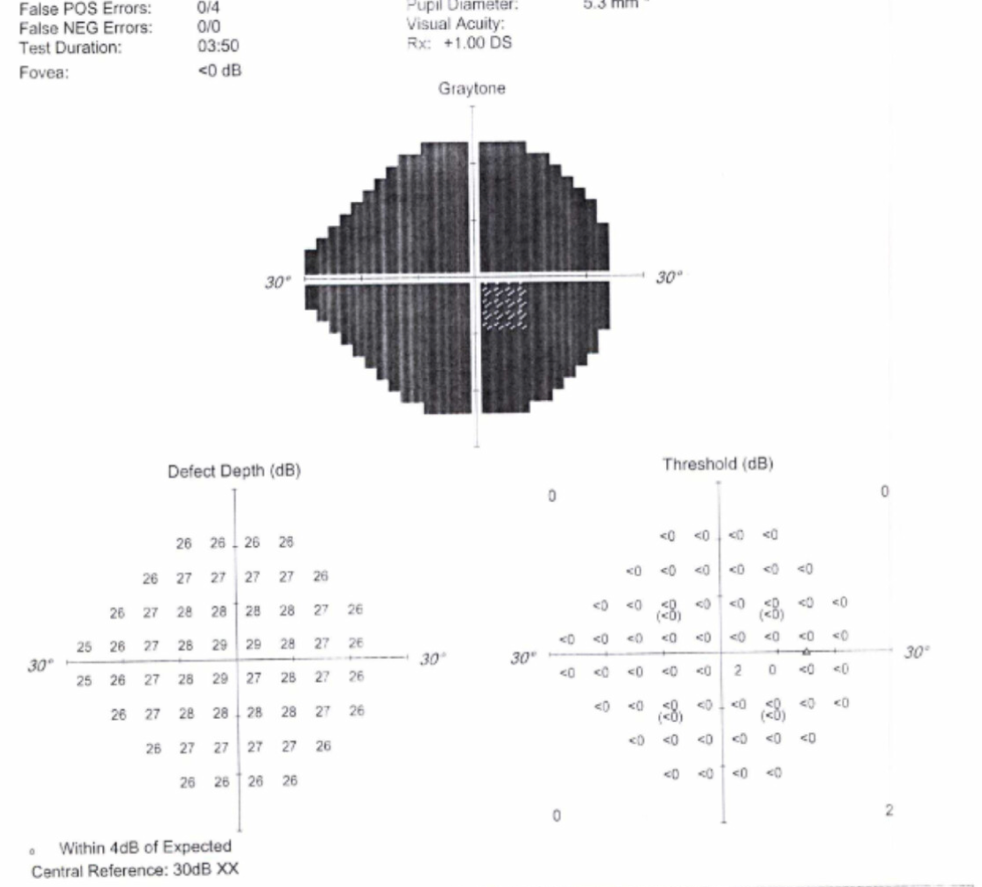

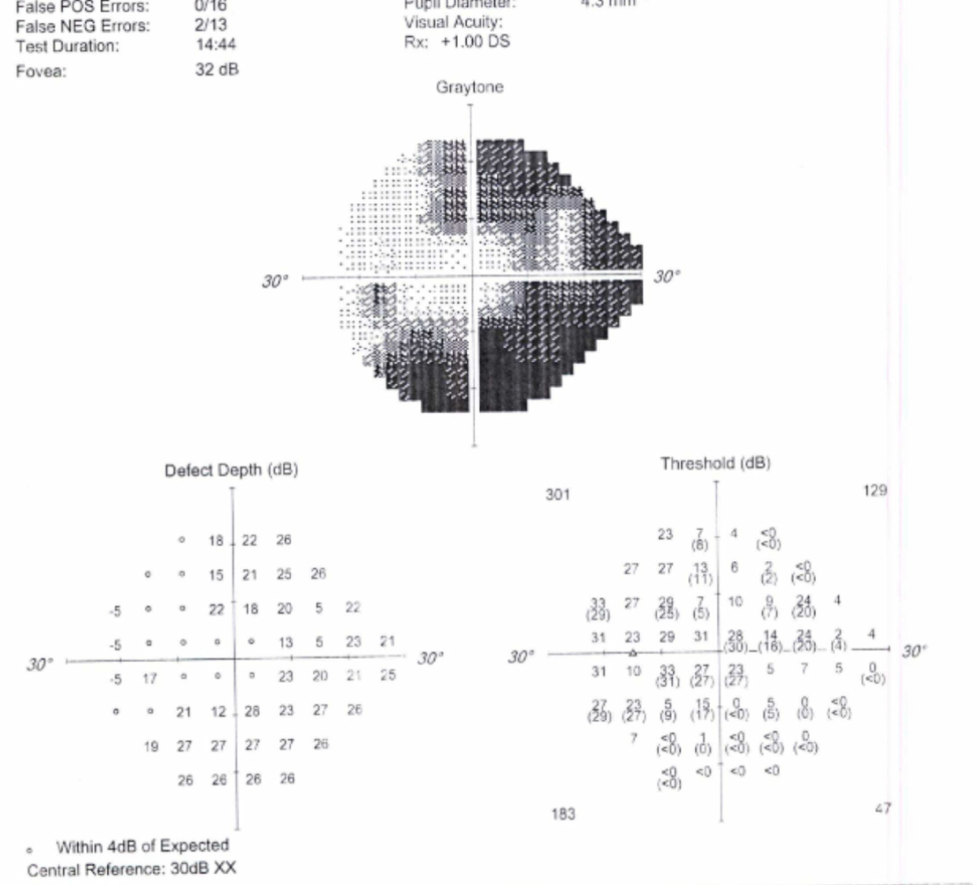


Supplementary Figure 2: Case 2

A. Case 2. Initial Humphrey visual field (HVF) 24-2 size 3 stimulus both eyes (OU) obtained 1 week after hospital discharge.


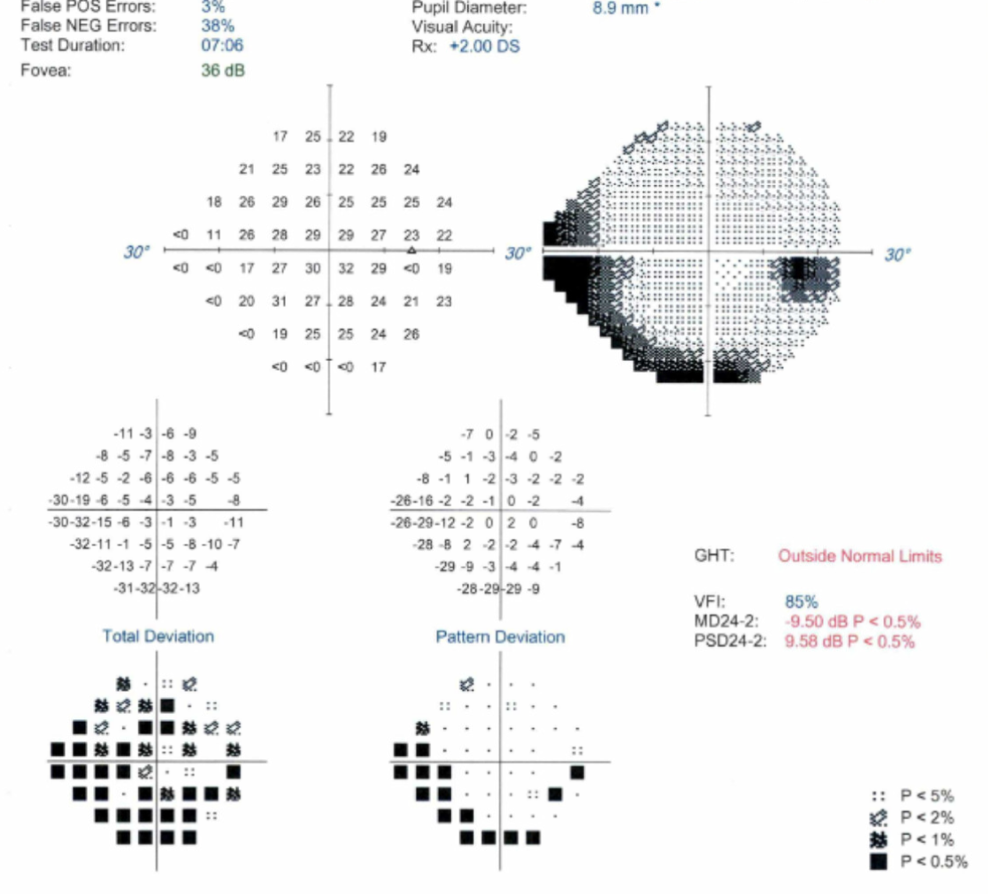

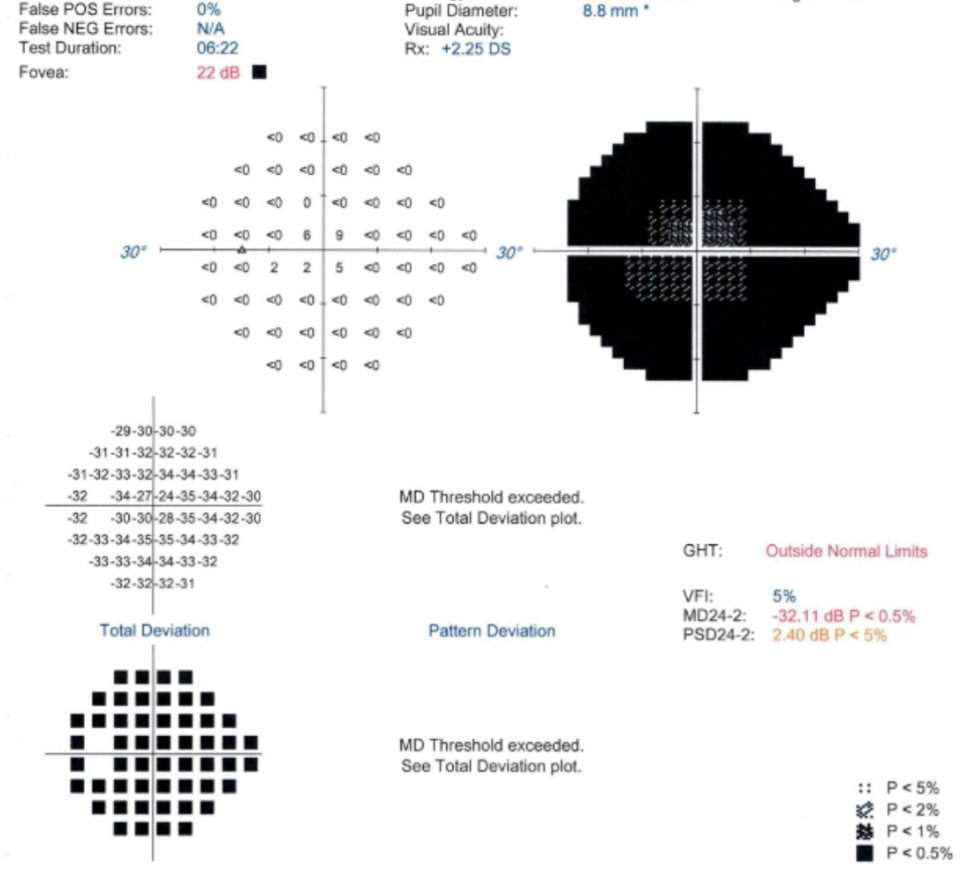


B. Case 2. HVF 24-2 with size 3 stimulus OU obtained 1 month after hospital discharge.


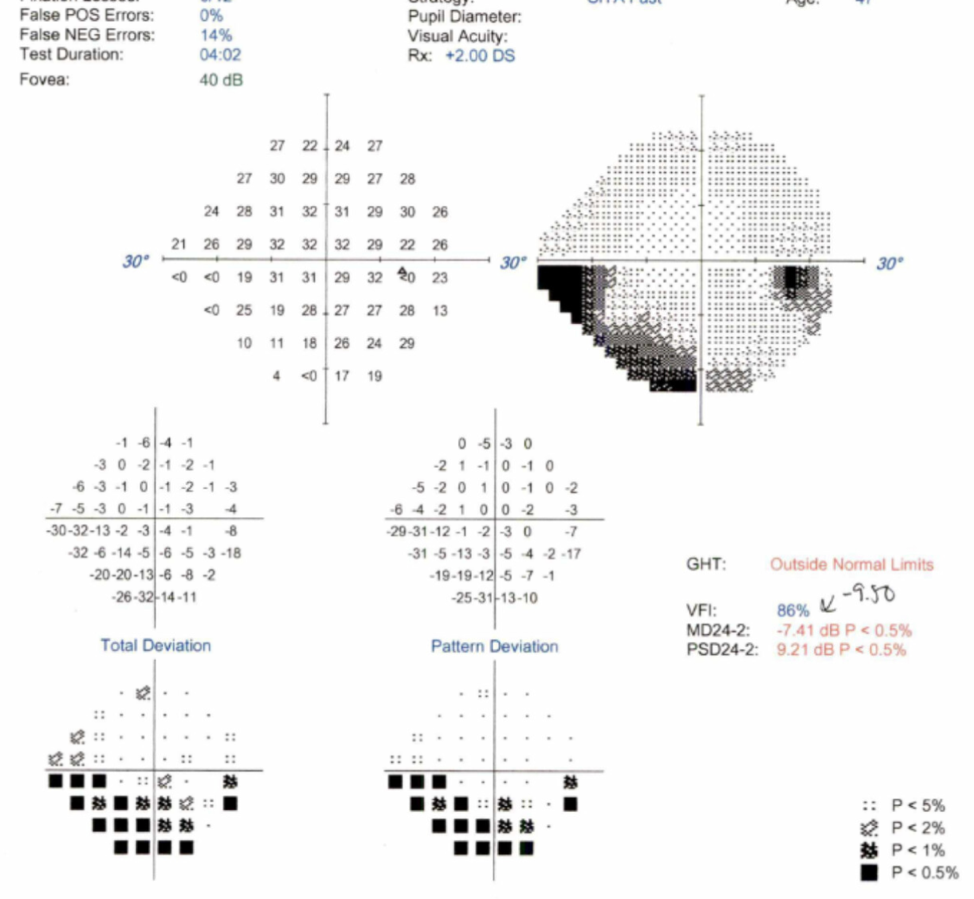

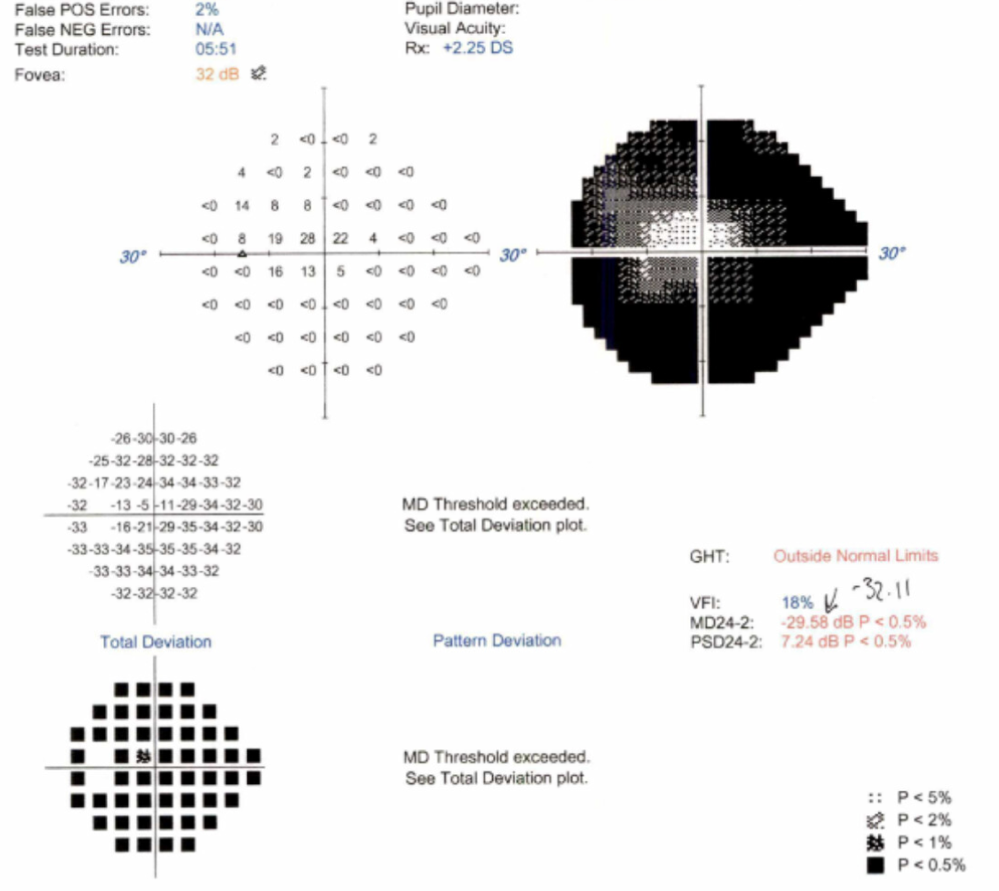


C. Worsening visual fields despite improved papilledema, HVF 24-2 size 3 stimulus OU.


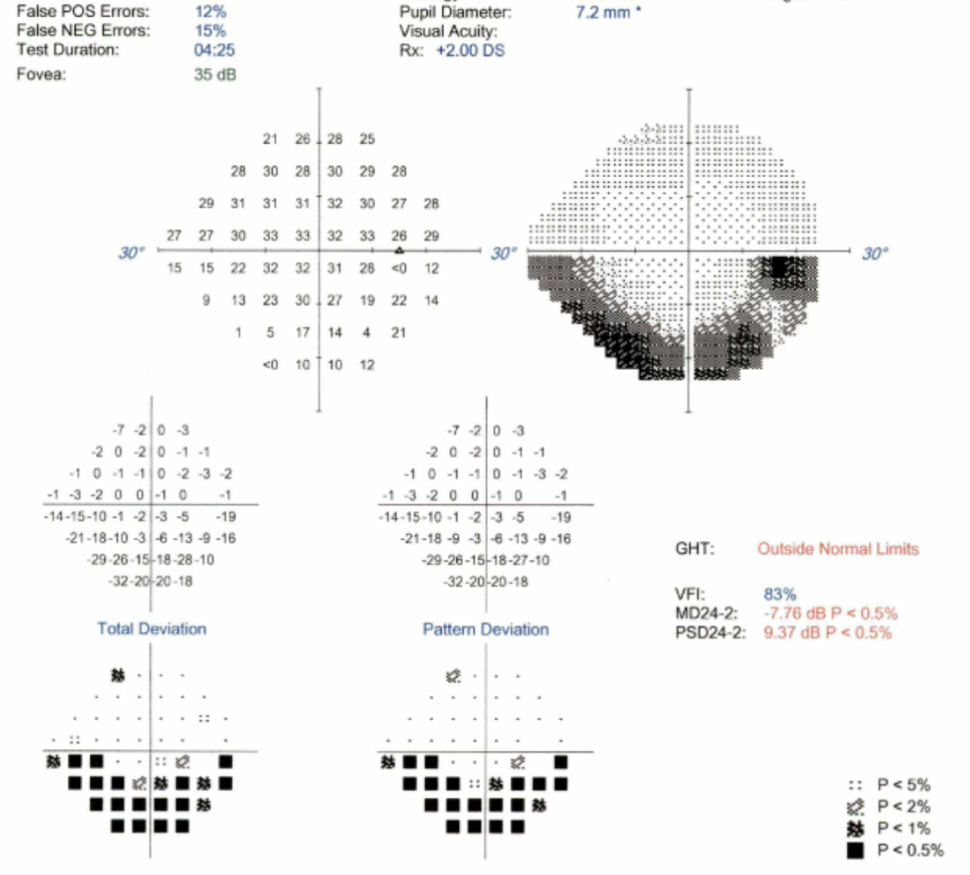

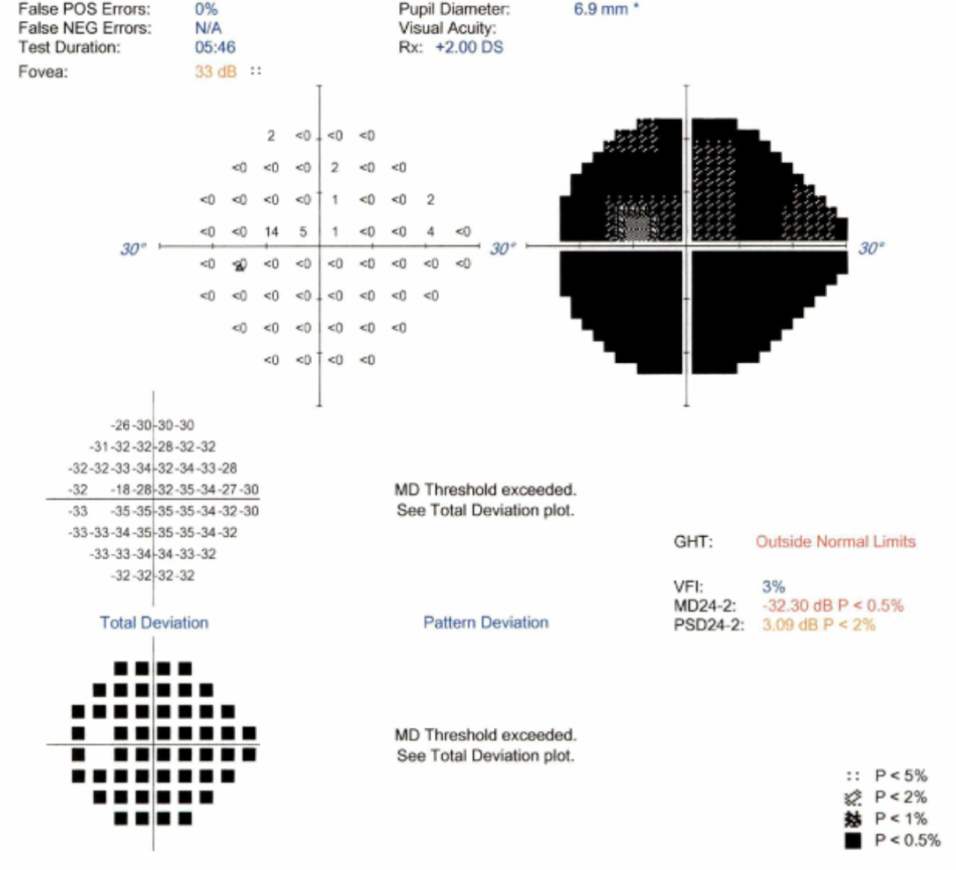


D. Most recent visual fields available, obtained around 1 year after initial presentation. HVF 24-2 with size 3 stimulus in right eye but size 5 stimulus in left eye.


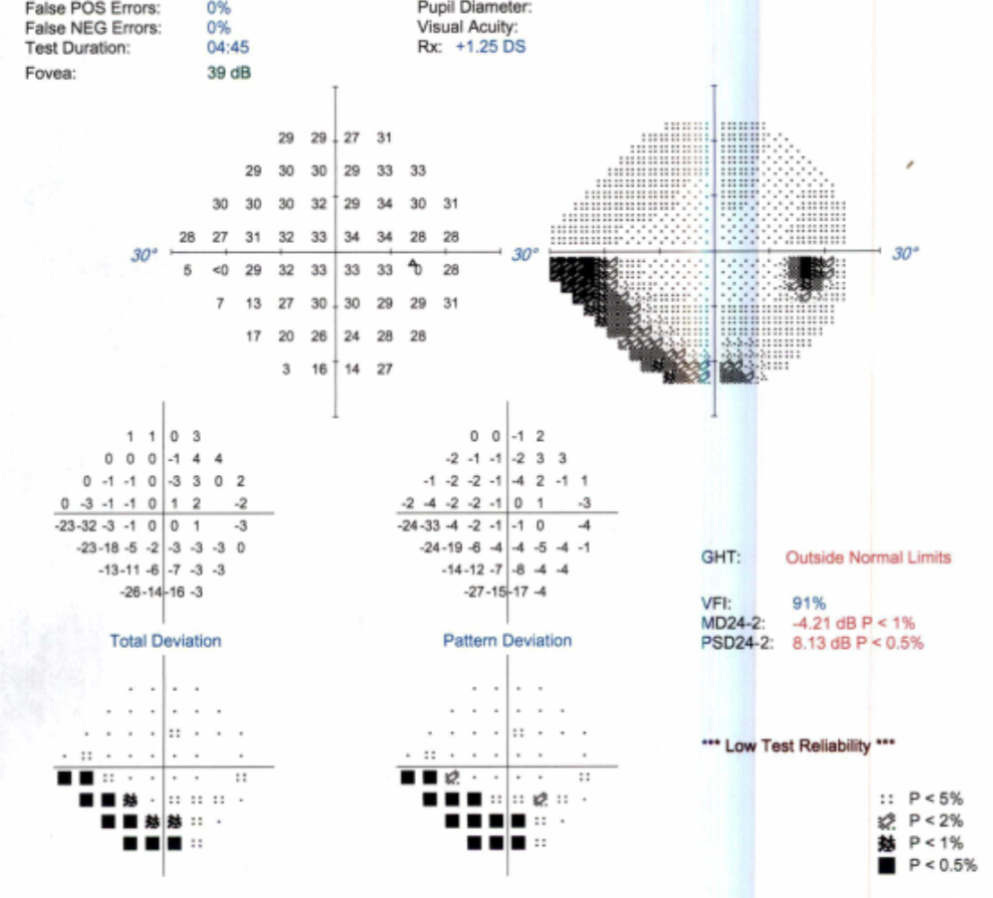

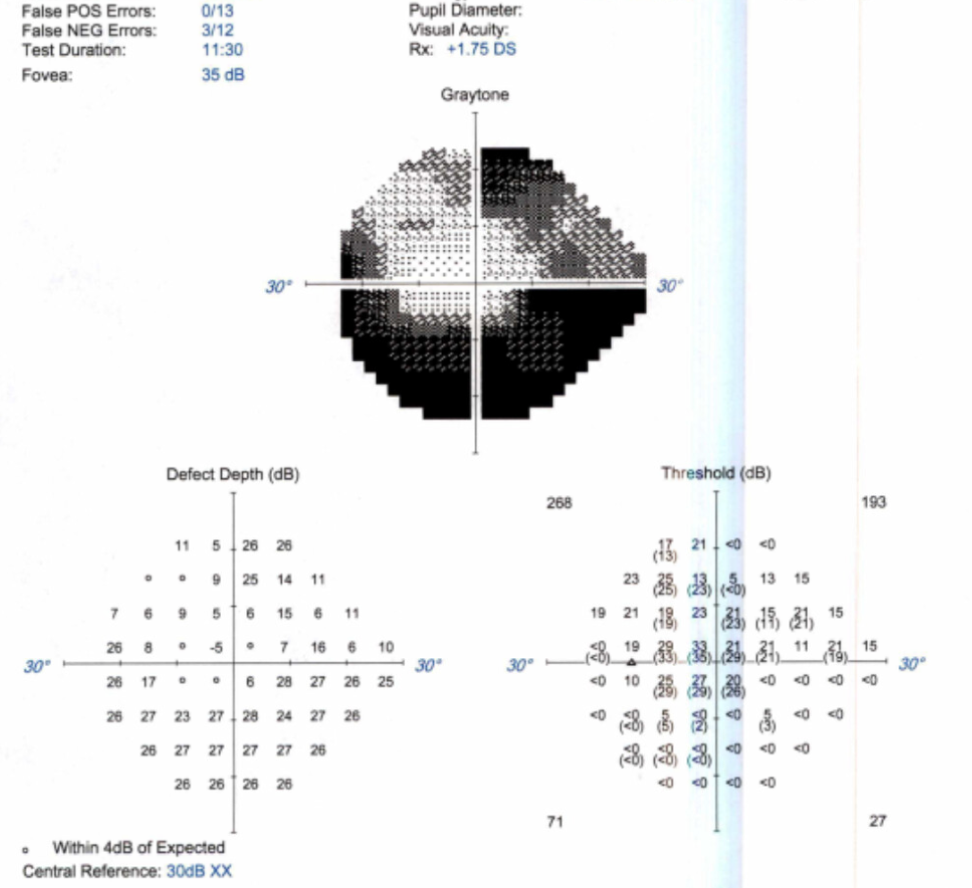

Supplement: Supplementary Figure 1 — Case 1: (A) Initial presentation Humphrey visual field (HVF) 24-2 size 3 stimulus both eyes (OU). (B) 2 weeks prior to ventriculoperitoneal (VP) shunt. HVF 24-2 size 3 stimulus OU. (C) Less than 2 weeks after VP shunt. HVF 24-2 size 3 stimulus OU. (D) Most recent visual field, obtained 3 years after initial presentation, with size 5 stimulus OU. [file DataSheet1.docx]
